# Early initiation of tolvaptan is associated with early discharge in patients with heart failure regardless of age

**DOI:** 10.1186/s12872-022-02640-7

**Published:** 2022-04-29

**Authors:** Shunsuke Kiuchi, Shinji Hisatake, Takayuki Kabuki, Takashi Oka, Shintaro Dobashi, Yoshiki Murakami, Takahide Sano, Takanori Ikeda

**Affiliations:** Department of Cardiovascular Medicine, Toho University Graduate School of Medicine, 6-11-1 Omori-nishi, Ota-ku, Tokyo, 143-8541 Japan

**Keywords:** Tolvaptan, The Length of hospital stay, Heart failure, Elderly

## Abstract

**Background:**

Elderly patients with heart failure (HF) have been observed to decrease activities of daily living (ADL) during hospitalization. Prevention of ADL decline from shortening of hospital stays is especially important in the elderly, because decreasing ADL is associated with poor prognosis. We investigated the relationship between the early initiation of tolvaptan (TLV) after hospitalization and the length of hospital stay in patients with HF aged younger than 80 years and aged 80 years and older.

**Methods:**

We analyzed 146 patients younger than 80 years (< 80) and 101 patients aged 80 years and older (≥ 80) who were hospitalized with HF from February 2011 to June 2016 and had initiated TLV. The relationship between the time until commencement of TLV and the length of hospital stay was assessed. Additionally, a comparison made between the TLV early start group (within the median) and the delayed start group (over the median) for both groups. Multivariate analysis was also performed on factors that required hospital stays below the median.

**Results:**

A significant correlation was observed between time to TLV initiation and the length of hospital stay (< 80: r = 0.382, *P* < 0.001; ≥ 80: r = 0.395, *P* < 0.001). The length of hospital stay in the early group was significantly longer than that in the delayed group for both groups (< 80: early 21.0 ± 13.0 days and 33.0 ± 22.7 days, respectively, *P* < 0.001; ≥ 80: early 21.3 ± 12.5 days and 32.9 ± 17.9 days, respectively, *P* < 0.001). Conversely, no statistically significant difference found in the length of hospital stay after initiation of TLV. Moreover, no increase in adverse events in the elderly observed. A multivariate analysis revealed that a predictive factor for short-term hospitalization was early administration of TLV regardless of age.

**Conclusions:**

The early initiation of TLV after hospitalization was associated with a shorter length of hospital stay in patients with HF regardless of age.

## Background

Tolvaptan (TLV), oral selective vasopressin type 2 receptor antagonists, approved in Japan in October 2010. In the United States and Europe, TLV used for hyponatremia and/or syndrome of inappropriate antidiuretic hormone. In Japan, TLV also became available for fluid retention due to heart failure (HF) [1]. Moreover, the V2 receptor antagonist effect of TLV has been suggested to have an effect on improving HF beyond its diuretic effect [2].

The prevalence of HF increases with aging. In Japan, which is facing an aging society, the number of patients with HF is expected to reach approximately 1.3 million in 2030 [3]. Cardiac death and re-admission for HF 1 year after discharge were reported to be approximately 10% and 30%, respectively [4]. Therefore, treatment focused on the prognosis is important in HF. Although HF reduced ejection fraction (EF) (HFrEF) and HF preserved EF (HFpEF) are classified as HF, cardio protective medications including β-blockers (BBs) and renin–angiotensin–aldosterone system (RAS) inhibitors are only useful with HFrEF [5]. The number of patients with HFpEF is expected to increase after 2030; however, these cardio protective medications are not recommended for HFpEF. One characteristic of patients with HFpEF is that they tend to be elderly, and it has been reported that guideline-based medical therapy might not be effective in patients with HFrEF older than 80 years of age [6]. In Japan, the percentage of elderly in inpatients with HF is on the rise [7], and HF treatment with an emphasis on activities of daily living (ADL) could be important, especially for patients with HF older than 80 years. Treatment for HF that shortens hospital stays is useful, especially in elderly patients, because prolonged hospital stays result in lower ADL. An association between the early administration of TLV and the length of hospital stay has been reported [8]; however, it is unclear whether early administration of TLV shortens the length of hospital stay in elderly patients with HF, and its safety is unclear. In the present study, we investigated the effectiveness of early initiation of TLV in patients with HF who were 80 years of age or older as well as that of those who were younger than 80 years.

## Material and methods

The ethics committee of Toho University Omori Medical Center approved the present study (approval number: M18271_17318). All experiments were performed in accordance with the Declaration of Helsinki. The present study was a retrospective, observational study at a single center. Therefore, we have posted the details of the study in opt-out format (granted a waiver of informed consent from study participants). Details about this study were disclosed on the website of Toho University Omori Medical Center and our department (Department of Cardiovascular Medicine) and the potential participants were given the opportunity to decline to be further enrolled in the study (opt-out). Due to retrospective nature of the present study, Toho University Omori Medical Center of the ethics committee granted a waiver for informed consent.

### Study participants

We enrolled 369 patients who were hospitalized for HF from February 2011 to June 2016 and started with TLV. HF was diagnosed according to the Framingham criteria, the American Heart Association guidelines, or the European Society of Cardiology guidelines. The exclusion criteria were as follows: died in hospital, transferred to another hospital, and a clinical scenario classification (CS) of 4 or 5. Ultimately, 146 patients younger than 80 years (< 80) and 101 patients 80 years of age and older (≥ 80) were investigated. The CS is a selection criterion to determine the treatment method in hospitalized patients with early-stage HF. It is typically decided by blood pressure (BP), except CS4 (acute coronary syndrome) and CS5 (isolated right HF) [9]. CS1-3 is decided as follows: CS1, elevated SBP (> 140 mm Hg); CS2, normal SBP (≥ 100 and ≤ 140 mm Hg); CS3, low SBP (< 100 mm Hg).

### Study outcomes

The main outcomes in both groups were the relationship between the time from hospitalization to commencement of TLV, and the length of hospital stay. We also investigated the time until commencement of TLV from hospitalization. These times were compared between the early use group (within the median day of TLV initiation) and the delayed use group (more than the median day of TLV initiation), in both the ≥ 80 group and the < 80 group. The findings in the ≥ 80 group were compared with those in the < 80 group.

Moreover, the patient characteristics between early and delayed commencement of TLV in both the ≥ 80 group and the < 80 group were compared. Multivariate analysis was also performed on factors that required hospital stays below the median.

We also investigated the safety of TLV (its adverse effects and whether or not it can be continued). For safety, hypernatremia, the incidence of serious liver and/or renal dysfunction, and adverse events (AEs) were investigated. Hypernatremia was defined as a serum sodium concentration below 150 mEq/L at baseline and above 150 mEq/L after treatment [10]. An AE was defined as any medical occurrence that was life threatening or that required inpatient hospitalization.

### Clinical profiles

We investigated levels of HF (New York Heart Association [NYHA] classification), the CS, medical history, and underlying heart disease. Hypertension, diabetes mellitus, dyslipidemia, chronic kidney disease, and prior HF hospitalization were assessed for the medical history. Underlying heart disease was classified into ischemic cardiomyopathy (ICM), hypertensive heart disease (HHD), valvular heart disease (VHD), tachycardia-induced cardiomyopathy (TIC), dilated cardiomyopathy (DCM), and others.

Additionally, we investigated age, gender, height, weight, and body mass index (weight [kg]/height^2^ [m^2^]).

### Concomitant medications

Concomitant medications were also investigated. We recorded the TLV administration rate and the dosage at discharge, as well as the presence or absence of combined RAS inhibitors at the start of TLV. For diuretics, we investigated the administration rates and the dosage of loop diuretics. The dose of loop diuretic was calculated in terms of furosemide (furosemide 20 mg is equivalent to azosemide 30 mg). Patients receiving loop diuretics had been receiving furosemide or azosemide. As for cardio protective medications, the administration rates of RAS inhibitors and BBs at discharge were recorded. An RAS inhibitor was defined as angiotensin converting enzyme inhibitor (in the present study, patients had been receiving imidapril, perindopril, or enalapril) or angiotensin II type1a receptor blocker (in the present study, patients had been receiving candesartan, valsartan, irbesartan, azilsartan, losartan, olmesartan, or telmisartan).

### Physiological function examinations

We measured systolic BP and diastolic BP (dBP) using an aneroid sphygmomanometer on admission and at discharge. The heart rate (HR) was measured in the supine position by standard 12-lead electrocardiography on admission.

Transthoracic echocardiography was investigated by 2 blinded physicians. We assessed left ventricular systolic function (EF), which was calculated using the Teichholz method using the parasternal long-axis view, or modified Simpson’s method by an apical 2- or 4-chamber view [11]. Patients were classified into HFrEF and HFpEF as per the Japan Circulation Society [5], and HFpEF was defined as EF > 50% [12]. The proportions of HFrEF and HFpEF were also evaluated.

### Laboratory data and chest X-ray

We measured C-reactive protein, electrolytes (sodium and potassium), liver function, renal function, hemoglobin, and brain natriuretic peptide (BNP). For liver function, aspartate aminotransferase, alanine aminotransferase, and lactate dehydrogenase were measured. Similarly, to determine renal function, we investigated blood urea nitrogen, creatinine, and the estimated glomerular filtration rate (eGFR). The eGFR was calculated according to the Japanese Society of Nephrology criteria: eGFR = 194 × Cr − 1.094 × age − 0.287 for men and 194 × Cr − 1.094 × age − 0.287 × 0.739 for women [13].

Physicians calculated the cardiothoracic ratio from chest X-ray film utilizing the maximal cardiac diameter and the intrathoracic diameter in a blinded manner.

### Statistical analysis

The continuous variables were expressed as mean ± standard deviation. We compared the groups employing an unpaired Student’s *t*-test. A probability (*p*) value of less than 0.05 was considered to indicate statistical significance. A Windows computer (Excel, Microsoft XP) and EZR (Saitama Medical Center, Jichi Medical University) were used, which is a graphical user interface for R version 2.13.0 (The R Foundation for Statistical Computing, Vienna, Austria) [14].

## Results

### Comparison of characteristics between patients 80 years and older and patients younger than 80

The clinical profiles of the ≥ 80 patients were compared with those patients < 80 (Table 1). In the elderly, there were many women with a physique. No statistically significant differences in medical history were observed between the 2 groups. NHYA and CS classifications were similar in the 2 groups.

**Table 1:** Patients’ clinical profiles

	< 80 years old	≥ 80 years old	*P* value
	(n = 146)	(n = 101)	
Age (years)	67.5 ± 10.0	85.1 ± 3.7	< 0.001
Male (n, %)	94, 64.4	48, 47.5	< 0.001
Height (cm)	160.5 ± 9.0	154.9 ± 8.6	< 0.001
Weight (kg)	56.5 ± 13.5	46.9 ± 9.5	< 0.001
Body mass index (kg/m^2^)	21.8 ± 4.1	19.5 ± 3.4	< 0.001
NYHA class (II/III/IV)	1/122/23	1/88/12	0.376
CS class (I/II/III)	48/83/15	25/66/10	0.270
Hypertension (n, %)	112, 76.7	82, 81.2	0.402
Diabetes mellitus (n, %)	37, 25.3	23, 22.8	0.645
Dyslipidemia (n, %)	29, 19.9	26, 25.7	0.277
Chronic kidney disease (n, %)	18, 12.3	20, 19.8	0.110
Prior HF hospitalization (n, %)	54, 37.0	45, 44.6	0.234

NYHA: New York Heart Association, CS: clinical scenario, HF: heart failure. Continuous data are expressed as the mean ± standard deviation or error. *P*-values were determined using the unpaired *t*-test

Underlying heart diseases in the elderly were ICM (40 patients, 27.4%), HHD (25 patients, 17.1%), VHD (27 patients, 18.5%), TIC (23 patients, 15.8%), DCM (20 patients, 13.7%), and others (11 patients, 15.8%). Similarly, ICM (39 patients, 38.6%), HHD (6 patients, 5.9%), VHD (36 patients, 35.6%), TIC (12 patients, 11.9%), DCM (1 patient, 0.9%), and others (7 patients, 6.9%) were found in the < 80 group. No significant differences were observed between the 2 groups.

In a laboratory analysis, no significant difference was found in the BNP (Table 2). Conversely, significant differences were found in dBP on admission and at discharge, HR, hemoglobin, and EF between the 2 groups, and the older elderly population accounted for more patients with HFpEF than the younger elderly population.

**Table 2:** Patient characteristics

	< 80 years old	≥ 80 years old	*P* value
	(n = 146)	(n = 101)	
Systolic BP (mm Hg) on admission	130.2 ± 29.7	127.1 ± 22.0	0.365
Diastolic BP (mm Hg) on admission	73.0 ± 20.2	67.8 ± 13.3	0.025
Heart rate (bpm)	92.1 ± 26.6	83.7 ± 21.8	0.009
Systolic BP (mm Hg) at discharge	107.0 ± 16.4	110.8 ± 14.5	0.066
Diastolic BP (mm Hg) at discharge	60.0 ± 9.3	57.6 ± 7.5	0.035
Sodium (mg/dl)	138.3 ± 4.2	138.6 ± 4.6	0.534
Potassium (mg/dl)	4.1 ± 0.6	4.2 ± 0.7	0.163
AST (IU/L)	67.6 ± 17.5	36.8 ± 3.4	0.148
ALT (IU/L)	56.4 ± 16.5	27.0 ± 3.6	0.144
LDH (IU/L)	355.0 ± 22.1	297.8 ± 10.1	0.041
BUN (mg/dl)	29.8 ± 17.9	32.0 ± 20.6	0.367
Creatinine (mg/dL)	1.43 ± 0.85	1.44 ± 0.92	0.991
eGFR (ml/min/1.73 m^2^)	46.4 ± 2.1	42.5 ± 2.0	0.189
Hemoglobin (g/dL)	12.3 ± 2.7	11.2 ± 2.0	< 0.001
Brain natriuretic peptide (pg/mL)	1325.8 ± 145.7	1244.0 ± 136.9	0.696
CTR (%)	63.2 ± 6.6	64.9 ± 6.2	0.039
Ejection fraction (%)	44.3 ± 18.5	52.6 ± 18.2	0.001
HFpEF (n, %)	56, 38.4	53, 52.5	0.028

BP: Blood pressure, AST: aspartate aminotransferase, ALT: alanine aminotransferase, LDH, lactate dehydrogenase, BUN: blood urea nitrogen, eGFR: estimated glomerular filtration rate, CTR: cardiothoracic ratio, HFpEF: heart failure preserved ejection fraction. Continuous data are expressed as the mean ± standard deviation or error. *P*-values were determined using the unpaired *t*-test

Baseline characteristics between the early and delayed commencement of TLV in both the ≥ 80 group and the < 80 group are shown in Tables 3 and 4. In the group older than 80 years, there were significantly differences in CS, BP and BNP. However, there were no statistically differences in the group younger than 80 years.

**Table 3:** Patient characteristics in the group older than 80 years

	Early use of TLV	Delayed use of TLV	*P* value
	(n = 40)	(n = 61)	
Body mass index (kg/m^2^)	19.4 ± 2.9	19.7 ± 4.2	0.603
NYHA class (II/III/IV)	0/55/6	1/33/6	0.705
CS class (I/II/III)	19/38/4	6/28/6	0.026
Systolic BP (mm Hg) on admission	130.8 ± 22.7	121.5 ± 19.9	0.037
Diastolic BP (mm Hg) on admission	70.3 ± 13.8	64.1 ± 11.5	0.020
Heart rate (bpm)	82.5 ± 20.5	85.5 ± 23.7	0.506
AST (IU/L)	34.0 ± 22.8	40.9 ± 47.0	0.329
ALT (IU/L)	24.1 ± 18.2	31.5 ± 53.4	0.323
eGFR (ml/min/1.73 m^2^)	43.8 ± 19.4	40.5 ± 22.8	0.431
Hemoglobin (g/dL)	11.4 ± 1.9	11.0 ± 2.1	0.326
Brain natriuretic peptide (pg/mL)	1502.2 ± 211.9	850.2 ± 96.8	0.019
Ejection fraction (%)	51.0 ± 18.1	55.1 ± 18.5	0.268
HFpEF (n, %)	30, 49.2	23, 57.5	0.418
Length of hospital stay	21.3 ± 12.5	32.9 ± 17.9	< 0.001
Time until commencement of TLV from hospitalization	3.0 ± 1.3	13.6 ± 11.6	< 0.001
Length of hospital stay after initiation of TLV	18.3 ± 12.4	19.3 ± 12.7	0.699

NYHA: New York Heart Association, CS: clinical scenario, BP: blood pressure, AST: aspartate aminotransferase, ALT: alanine aminotransferase, eGFR: estimated glomerular filtration rate, HFpEF: heart failure preserved ejection fraction, TLV: tolvaptan. Continuous data are expressed as the mean ± standard deviation or error. *P*-values were determined using the unpaired *t*-test

**Table 4:** Patient characteristics in the group younger than 80 years

	Early use of TLV	Delayed use of TLV	*P* value
	(n = 79)	(n = 67)	
Body mass index (kg/m^2^)	21.7 ± 3.9	21.9 ± 4.2	0.778
NYHA class (II/III/IV)	1/68/10	0/54/13	0.203
CS class (I/II/III)	26/46/7	23/36/8	0.760
Systolic BP (mm Hg) on admission	131.7 ± 28.0	128.6 ± 31.7	0.533
Diastolic BP (mm Hg) on admission	69.7 ± 22.2	75.7 ± 17.9	0.073
Heart rate (bpm)	89.0 ± 23.5	94.7 ± 28.8	0.195
AST (IU/L)	62.4 ± 24.5	73.6 ± 24.9	0.750
ALT (IU/L)	51.6 ± 21.8	62.0 ± 25.2	0.754
eGFR (ml/min/1.73 m^2^)	49.0 ± 26.5	43.3 ± 21.5	0.156
Hemoglobin (g/dL)	12.6 ± 2.2	12.0 ± 3.2	0.229
Brain natriuretic peptide (pg/mL)	1153.2 ± 118.0	1529.3 ± 284.8	0.199
Ejection fraction (%)	45.3 ± 17.6	43.2 ± 19.5	0.509
HFpEF (n, %)	31, 39.2	25, 37.3	0.813
Length of hospital stay	21.0 ± 13.0	33.0 ± 22.7	< 0.001
Time until commencement of TLV from hospitalization	2.5 ± 1.0	12.7 ± 13.3	< 0.001
Length of hospital stay after initiation of TLV	18.6 ± 13.1	20.3 ± 16.9	0.479

NYHA: New York Heart Association, CS: clinical scenario, BP: blood pressure, AST: aspartate aminotransferase, ALT: alanine aminotransferase, eGFR: estimated glomerular filtration rate, HFpEF: heart failure preserved ejection fraction, TLV: tolvaptan. Continuous data are expressed as the mean ± standard deviation or error. *P*-values were determined using the unpaired *t*-test

### Tolvaptan and heart failure in the study participants

**Fig. 1:**
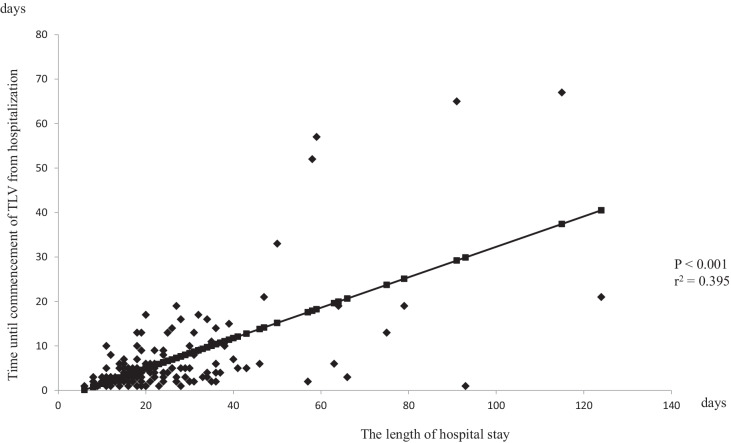
A regression curve of the relationship between the length of hospital stay and time until commencement of TLV from hospitalization in patients with heart failure 80 years of age and older. Time until commencement of TLV from hospitalization was strongly associated with the length of hospital stay (*P* < 0.001, r^2^ = 0.395)

**Fig. 2:**
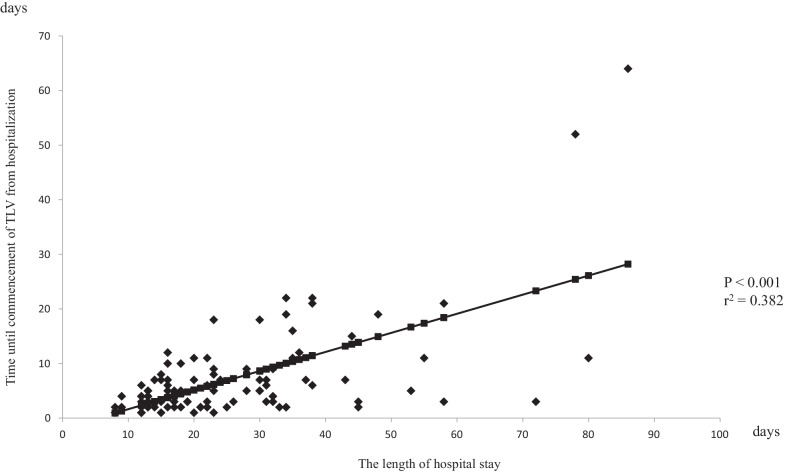
A regression curve of the relationship between the length of hospital stay and time until commencement of TLV from hospitalization in patients with heart failure younger than 80 years. Time until commencement of TLV from hospitalization was strongly associated with the length of hospital stay (*P* < 0.001, r^2^ = 0.382)

The association between the length of hospital stay and the time until commencement of TLV from hospitalization is illustrated in Fig. 1 (for ≥ 80) and Fig. 2 (for < 80). Both indicated a significant positive correlation.

The median of the time until initiation of TLV from hospitalization in the ≥ 80 and < 80 groups were 5 and 4 days, respectively. In the ≥ 80 group, the length of hospital stay and time until commencement of TLV from hospitalization in the early use group were significantly shorter than those of the delayed use group (the length of hospital stay: 21.3 ± 12.5 and 32.9 ± 17.9, respectively, *P* < 0.001; time until commencement of TLV from hospitalization: 3.0 ± 1.3 and 13.6 ± 11.6, respectively, *P* < 0.001) (Table 3). These results were similar in the < 80 group (the length of hospital stay: 21.0 ± 13.0 and 33.0 ± 22.7, respectively, *P* < 0.001; time until commencement of TLV from hospitalization: 2.5 ± 1.0 and 12.7 ± 13.3, respectively, *P* < 0.001) (Table 4). On the other hand, there were no significant differences in the length of hospital stay after initiation of TLV in either group (≥ 80 group: 18.3 ± 12.4 and 19.3 ± 12.7, *P* = 0.699; < 80 group: 18.6 ± 13.1 and 20.3 ± 16.9, *P* = 0.479) (Tables 3 and 4).

In addition, a multivariate analysis revealed that a predictive factor for short-term hospitalization in the ≥ 80 group was early administration of TLV (Table 5). These results were similar in the < 80 group (Table 6).

**Table 5:** Multivariate analysis for prediction of short-term hospitalization in age group older than 80 years

	HR	*p* value	HR	*p* value	HR	*p* value
Early administration of TLV	1.280	< 0.001	1.230	< 0.001	1.250	< 0.001
Age (years)	0.895	0.089				
BMI (kg/m^2^)	0.985	0.820				
Sex (men)	0.548	0.190				
Clinical scenario 1			1.300	0.697		
Systolic BP (mm Hg)			1.000	0.877		
NHYA					0.441	0.268
BNP (pg/dl)					1.000	0.595
Hemoglobin (g/dl)					0.893	0.346

TLV: Tolvaptan, BMI: body mass index, BP: blood pressure, NHYA: New York Heart Association Classification, BNP: brain natriuretic peptide. The multivariate analysis was performed applying Cox proportional hazards models

**Table 6:** Multivariate analysis for prediction of short-term hospitalization in the group younger than 80 years

	HR	*p* value	HR	*p* value	HR	*p* value
Early administration of TLV	1.210	< 0.001	1.200	< 0.001	1.190	< 0.001
Age (years)	0.998	0.904				
BMI (kg/m^2^)	0.925	0.104				
Sex (men)	0.960	0.918				
Clinical scenario 1			1.310	0.595		
Systolic BP (mm Hg)			1.000	0.919		
NHYA					2.040	0.159
BNP (pg/dl)					1.000	0.588
Hemoglobin (g/dl)					0.917	0.205

TLV: tolvaptan, BMI: body mass index, BP: blood pressure, NHYA: New York Heart Association Classification, BNP: brain natriuretic peptide. The multivariate analysis was performed applying Cox proportional hazards models

Comparing the 2 groups, no significant differences were observed in the administration rate or the dosage of TLV at discharge in the ≥ 80 group compared with the < 80 group: the administration rate of TLV was 47 (46.5%) patients vs 73 (50%) patients, respectively, *P* = 0.594; the dosage of TLV was 7.33 ± 3.92 mg/day vs 8.30 ± 4.70 mg/day, respectively, *P* = 0.092, respectively) (Table 7).

**Table 7:** Patients’ concomitant medications

	< 80 years old	≥ 80 years old	*P* value
	(n = 146)	(n = 101)	
Administration rate of TLV at discharge (n, %)	83, 50.0	47, 46.5	0.594
Dosage of TLV at discharge (mg/day)	8.30 ± 4.70	7.33 ± 3.92	0.092
Presence rate of RAS-I combined use at the start of TLV (n, %)	107, 73.3	83, 82.2	0.104
Administration rate of loop diuretics at discharge (n, %)	111, 76.0	82, 81.2	0.337
Dosage of loop diuretics at discharge (mg/day)	29.1 ± 23.3	24.5 ± 14.0	0.115
Administration rate of beta-blockers at discharge (n, %)	122, 83.6	80, 79.2	0.386
Administration rate of ACE-Is/ARBs at discharge (n, %)	90, 61.6	57, 56.4	0.414

TLV: Tolvaptan, ACE-I: angiotensin converting enzyme inhibitor, ARB: angiotensin II receptor blocker. Continuous data are expressed as the mean ± standard deviation. *P*-values were determined using the unpaired *t*-test

### Concomitant medications between each group

In a comparison between the ≥ 80 and < 80 groups, there were no significant differences in TLV-related findings (Table 7). Similarly, loop diuretic-related findings showed no significant differences; however, the dosage of diuretics (TLV and loop diuretics) was lower in the ≥ 80 than the < 80 group. No statistical difference was observed in cardio protective medications between the groups.

### Safety of tolvaptan

Of 247 patients, hypernatremia incidence was observed in 4 (1.62%) patients. Two (2.0%) patients were included in the ≥ 80 group. Another 2 (1.4%) patients were included in the < 80 group, and there were no statistical differences between the groups. No case of serious hypernatremia or central pontine myelinolysis was observed. Moreover, no case of serious liver or renal dysfunction was reported. Serious AEs were not detected.

## Discussion

### Comparison of baseline characteristics in heart failure

The EF in the ≥ 80 group was statistically higher than that in the < 80 group, and the proportion of HFpEF in the ≥ 80 s group was significantly higher than that in the < 80 group. These results were consistent with the characteristics of HF among the elderly in Japan. A previous study had reported that patients with HFpEF included more elderly and women, and they had anemia. Conversely, patients with HFrEF had high heart rates [15]. The participants in the present study included patients with backgrounds that matched several of these characteristics. In addition, the length of hospital stay for HF in Japan is reported to be approximately 20 days [16]. Therefore, the present study reflected the real world experience of HF, although only those who received TLV were included.

### The length of hospital stays in elderly patients with heart failure

Bed rest and oxygen administration are necessary for the treatment of HF; however, these often cause a decrease in the ADL of elderly patients hospitalized with HF. Long-term bed rest has been associated with increased in-hospital death from HF [17], and the prognosis of HF worsens when physical activity decreases during hospitalization [18]. Particularly, muscle weakness in elderly patients during hospitalization is a factor of poor prognosis comparable to HF; therefore, shortened bed rest and early discharge are important [19]. The early initiation of TLV has been reported to shorten bed rest in elderly patients with HF [20]. In the present study, the early initiation of TLV shortened the length of hospital stay even among patients 80 years and older, and a significant positive correlation found between time until initiation of TLV from hospitalization and the length of hospital stay. Additionally, only early administration of TLV was predicted to shorten hospitalization in both the over and < 80 groups.

### Relationship between diuretics and the prognosis of elderly patients with heart failure

No significant difference found in the administration rate of TLV at discharge regardless of age, and approximately 50% of the patients continued TLV. Early initiation of TLV reported to be associated with the prognosis after discharge in patients requiring continued TLV [21]. Moreover, the administration of a higher dosage of loop diuretics could exacerbate HF [22]. These results are the same in elderly patients, and diuretics usage has been reported to be a prognostic factor in patients with HF 80 years and older. [23] In the present study, the dosage of diuretics (TLV and loop diuretics) was lower in the ≥ 80 than the < 80 group, although there was no significant difference.

### Safety of tolvaptan in heart failure patients older than 80 years

TLV can easily maintain renal hemodynamics and renal function, because TLV mainly removes water from the third space [24]. However, high TLV dosage has been reported to worsen renal function [25]. In the present study, the early initiation of TLV did not increase the dosage significantly. Moreover, TLV post-marketing surveillance has reported some physician-reported adverse drug reactions [26]. No AEs were observed in either group in the present study. TLV has also been reported to be used without increased AEs, even in patients older than 85 years [27]. Therefore, TLV can be used relatively safely even in older patients with HF with careful follow-up.

### Study limitations

The present study was a single-center, retrospective, observational study. The attending physicians determined the use of TLV, because the study was designed from the retrospective, not interventional, perspective. The choice of medications could have been influenced by the patients’ baseline characteristics. Additionally, the manner in which TLV is used changes from year to year. Tolvaptan’s prescription rate has increased, and the time until commencement of TLV from hospitalization has shortened year by year [28]. The continuation of TLV at discharge has also been reported to contribute to the improvement in prognoses [29], and the continuation after discharge is increasing annually. These potential biases could have affected the results. Therefore, further large, prospective, clinical studies are required to confirm these results. In addition, ADL was not evaluated, and the relationship between ADL and length of hospital stay was unclear, because the present study was a retrospective study. ADL before administration of TLV was also unclear.

## Conclusion

The early initiation of Tolvaptan was associated with a shorter length of hospital stay in patients with HF, both in those aged younger than 80 years and in those aged 80 years and older.

## Data Availability

The datasets generated and/or analysed during the current study are not publicly available due additional analysis is currently underway but are available from the corresponding author on reasonable request.

## Abbreviations

TLV: Tolvaptan
HF: Heart failure
HFrEF: Heart failure reduced ejection fraction
HFpEF: Heart failure preserved ejection fraction
BB: β-Blocker
RAS: Renin–angiotensin–aldosterone system
ADL: Activities of daily living
AE: Adverse event
NHYA: New York Heart Association classification
CS: Clinical scenario classification
ICM: Ischemic cardiomyopathy
HHD: Hypertensive heart disease
VHD: Valvular heart disease
TIC: Tachycardia-induced cardiomyopathy
DCM: Dilated cardiomyopathy
BMI: Body mass index
sBP: Systolic blood pressure
dBP: Diastolic blood pressure
HR: Heart rate
TEE: Transthoracic echocardiography
EF: Ejection fraction
Hb: Hemoglobin
BNP: Brain natriuretic peptide
BUN: Blood urea uitrogen
Cre: Creatinine
eGFR: Estimated glomerular filtration rate
